# Seasonal Effects of *UCP1* Gene Polymorphism on Visceral Fat Accumulation in Japanese Adults

**DOI:** 10.1371/journal.pone.0074720

**Published:** 2013-09-25

**Authors:** Kazuhiro Nakayama, Hiroshi Miyashita, Yoshiko Yanagisawa, Sadahiko Iwamoto

**Affiliations:** 1 Division of Human Genetics, Center for Molecular Medicine, Jichi Medical University, Shimotsuke-shi, Tochigi, Japan; 2 Jichi Medical University Health Care Center, Shimotsuke-shi, Tochigi, Japan; Wake Forest School of Medicine, United States of America

## Abstract

Uncoupling protein 1 (*UCP1*) and β3 adrenergic receptor (*ADRB3*) genes play central roles in the thermogenesis of brown adipose tissue (BAT) in adult humans. However, the importance of single-nucleotide polymorphisms (SNPs) in both genes during the development of obesity is controversial. Although active BAT in adult humans is frequently observed in the winter season, the effects of sampling season have not been taken into consideration in previous association studies. Here, we tested the associations of *UCP1* -3826A/G and *ADRB3* Trp64Arg with body mass index (BMI) and visceral fat area (VFA) in 3013 Japanese adults sampled during different seasons. Association between SNPs and the obesity-related traits were assessed using multiple linear regression models, including sex, age, physical activity, and genotypes. Both SNPs did not show significant associations in the models based on the entire cohort. However, in subsets comprising individuals mainly sampled from winter to spring, *UCP1* showed significant associations with VFA (*P* = 0.0098) and VFA adjusted for BMI (*P* = 0.0128). Moreover, the effects of *UCP1* on VFA were strongly negatively correlated with outdoor temperature (*P = *0.00011), but not with night length (*P* = 0.039). *ADRB3* did not show these associations, but an additive effect with *UCP1* was observed for VFA adjusted for BMI (*P* = 0.0067). Subsets sampled in the hot season did not show significant associations for both SNPs. The season-specific effects of *UCP1* on VFA were consistent with a previous finding that active BAT was more frequently found in winter than in summer, and supported the importance of cold stress in BAT activation and the significance of BAT in the development of obesity in adult humans.

## Introduction

Brown adipose tissue (BAT) plays an important role in non-shivering thermogenesis in mammals and has received much attention as a potential target in the treatment of obesity. The presence of active BAT in neonatal humans is well documented, but active BAT was believed to be absent or to merely contribute to energy and thermoregulation in adult humans. Recent studies have provided evidence for the presence of active BAT in adult humans under cold-stress conditions [1]–[4]. The activity of BAT in adult humans is reduced with age, and inactive BAT is thought to be linked to reduced energy expenditure and age-induced obesity [5].

Uncoupling protein 1 (UCP1) and β3 adrenergic receptor (ADRB3) are key molecules involved in the thermogenesis of BAT. UCP1 is a mitochondrial membrane protein that mediates heat generation by enhancing proton conductivity of the inner lumen. The expression of UCP1 in BAT is regulated by the sympathetic nervous system and by ADRB3. Genes encoding UCP1 (*UCP1*) and ADRB3 (*ADRB3*) have been studied to elucidate genetic variations accounting for the susceptibility to obesity. Most association studies have focused on a regulatory single-nucleotide polymorphism (SNP) of *UCP1* (-3826A/G) and/or a coding SNP in *ADRB3* (Trp64Arg). Significant associations with various obesity-related traits, primarily body mass index (BMI), have been reported, but the results in several studies were controversial [6], [7]. Yoneshiro et al. recently revealed that these 2 SNPs are associated with age-related decreases in BAT activity in Japanese adults [8]; however, the roles of variations in *UCP1* and *ADRB3* on the development of obesity are still unclear.

The detection rate of BAT is influenced by season in addition to sex, age, and obesity status of individuals; BAT is more frequently detected in examinations conducted in the winter than those conducted in the summer, even in the same individual [4]. Nevertheless, previous association studies of *UCP1* and *ADRB3* did not take the effect of sampling season into consideration. Moreover, BMI may not be an optimal indicator of obesity since BMI is influenced by the weight of organs other than adipose tissues. Visceral fat has high lipogenic/lipolytic activities and can act as an energy reservoir, responding to relatively short-term energy balance. In fact, short-duration, moderate-intensity exercises could effectively suppress the accumulation of visceral fat, but not subcutaneous fat, in adult humans [9]. Therefore, visceral fat area, rather than BMI, was thought to be the superior phenotype for testing the associations of *UCP1*/*ADRB3* and obesity.

In the present study, we tested associations of these 2 SNPs with visceral fat accumulation in Japanese adults sampled during various seasons.

## Materials and Methods

The design of the present study was approved by the Ethics Committee of Jichi Medical University. The recruited individuals provided written informed consent. A total of 3013 Japanese individuals who attended general health checkups at the Jichi Medical University Hospital from January 2009 to March 2011 were included. Visceral fat area (VFA) was measured using the bioelectrical impedance analysis (BIA) method [10]. Consistency between the results obtained with the BIA and conventional computed tomography methods has been confirmed [11], [12]. The demographics and characteristics of the studied population are summarized in Table 1. Other details about the studied population were described in our previous paper [11].

**Table 1:** Characteristics of the studied population.

	Entire Cohort	January–April	July–October	
Numbers of individuals	3013	1080	979	
Percentage of males^1^	53.8	52.1	53.6	*P*>0.05
Age^2^	51.7±0.2	51.7±0.3	52.0±0.3	*P*>0.05
Body mass index (kg/m^2^)^2^	23.5±0.1	23.5±0.1	23.4±0.1	*P*>0.05
Waist circumstance (cm)^2^	85.0±0.2	84.9±0.3	84.7±0.3	*P*>0.05
Visceral fat area (cm^2^)^2^	90.5±0.8	89.4±1.3	89.6±1.4	*P>*0.05

1 Difference between January–April and July–October was assessed with χ^2^ test.

2 Means and standard errors are shown. Difference between January–April and July–October was assessed with Student's *t* test.

Genotyping of *UCP1* -3826A/G (rs1800592) and *ADRB3* Trp64Arg (rs4994) was performed using the TaqMan method. The Hardy-Weinberg equilibrium of the genotype was tested using the chi-square goodness of fit test. These SNPs were tested for associations with BMI, VFA, and VFA adjusted for BMI using multiple linear regression models. One hundred two individuals who had undergone the abdominal surgery were excluded from the analyses. Genotypes of each SNP were coded as 0, 1, or 2 according to numbers of the obesity-risk *UCP1* G allele and *ADRB3* Arg allele. Sex, age, and genotype were included as independent variables. To adjust for inter-individual variability of physical activity, speed of walking (slow = 0 and fast = 1), obtained by including this question in the health checkup questionnaire “Do you walk faster than other people of same sex and about same ages?”, was included as an independent variable. BMI was also included as an independent variable in models adjusted for BMI. Normalization of dependent variables was not applied since the distribution of BMI and VFA was not strongly skewed. Sex-specific multiple linear regression models included genotype, age, and speed of walking as independent variables. The significance level was set at 0.025, which corresponded to 0.05 in 2 independent tests. The summed number of risk alleles of 2 SNPs in each individual, which ranged from 0 to 4, was also used as an independent variable.

Correlations between the effects of *UCP1* -3826A/G on VFA and environmental factors were assessed using Spearman's rank correlation method. β coefficients obtained in the multiple linear regression models were used as variables representing the effects of *UCP1* -3826A/G on VFA. The β coefficients indicated the effect size of SNP on VFA (adjusted for age and sex) and larger positive β coefficients indicated carriers of the G allele that tended to accumulate more visceral fat. Monthly outdoor temperature and night length data measured at the nearest observatory were obtained from the Japan Meteorological Agency. The significance level of the correlation test was set to be *P*<0.05. The above-described statistical tests were performed using SPSS version 20 (IBM Corporation, NY).

## Results

Results of association analyses are summarized in Table 2. β coefficients and *P* values for independent variables other than genotypes are shown in Table S1. Frequencies of the *UCP1* G allele and *ADRB3* Arg allele in our population were 0.51 and 0.19, respectively. Both SNPs did not show deviations from the Hardy-Weinberg equilibrium (*P*>0.1). In multiple linear regression models based on all genotyped individuals, no significant associations with BMI, VFA, or VFA adjusted for BMI were observed for both SNPs (*P*>0.05). To test the season-dependent effects of *UCP1* and *ADRB3*, 2 subsets were drawn from the population (Tables 1 and 2). The first subset consisted of 1080 individuals sampled during the cold season (January through April), and the second subset consisted of 979 individuals sampled during the hot season (June through September). In the first subset, *UCP1* showed significant associations with VFA (*P* = 0.0197), and the G allele exhibited an increased VFA (GG genotype showed a 3.39±1.45-cm^2^ increase in VFA compared to AA genotype). *UCP1* was also associated with VFA adjusted for BMI (*P* = 0.0246), but did not associate with BMI (*P* = 0.203). In the second subset, however, *UCP1* did not show association with either VFA or VFA adjusted for BMI (*P = *0.667 and *P* = 0.954, respectively). *ADRB3* did not associate with any trait in both subsets. To know whether the associations with VFA or VFA adjusted for BMI were sex specific in nature, we constructed multiple linear regression models separately for male and female populations. The association between *UCP1* and VFA was significant for males in the entire cohort as well as in the cold season; however the association did not remain for females suggesting a sex-specific association (Table S2).

**Table 2:** Summary of the initial association analyses.

	BMI		VFA		a-VFA	
	β(S.E.)	*P*	β(S.E.)	*P*	β(S.E.)	*P*
Entire cohort (F = 1339, M = 1572)						
*UCP1* -3826 G (0.51)	0.011(0.018)	0.551	0.022(0.014)	0.106	0.015(0.008)	0.051
*ADRB3* 64Arg (0.19)	−0.037(0.018)	0.038	−0.018(0.014)	0.189	0.005(0.008)	0.521
January–April (F = 517, M = 563)						
*UCP1* -3826G (0.50)	0.037(0.029)	0.203	0.053(0.023)	0.0197^*^	0.029(0.013)	0.0246^*^
*ADRB3* 64Arg (0.20)	−0.033(0.029)	0.260	−0.006(0.023)	0.801	0.015(0.013)	0.242
July–October (F = 454, M = 525)						
*UCP1* -3826G (0.52)	0.018 (0.031)	0.667	0.010(0.024)	0.667	−0.001(0.014)	0.954
*ADRB3* 64Arg (0.19)	−0.066(0.031)	0.032	−0.045(0.023)	0.057	−0.004(0.014)	0.763

BMI: body mass index, VFA: visceral fat area, a-VFA: VFA adjusted for BMI, F: number of females, M: number of males. S.E.: standard error for β coefficient. Relative allele frequencies of the obesity risk alleles are shown in parenthesis. β coefficients and *P* values for the copy number of risk alleles in multiple linear regression models are shown. Asterisks indicate significant results after applying Bonferroni correction (2 independent tests).

**Figure 1:**
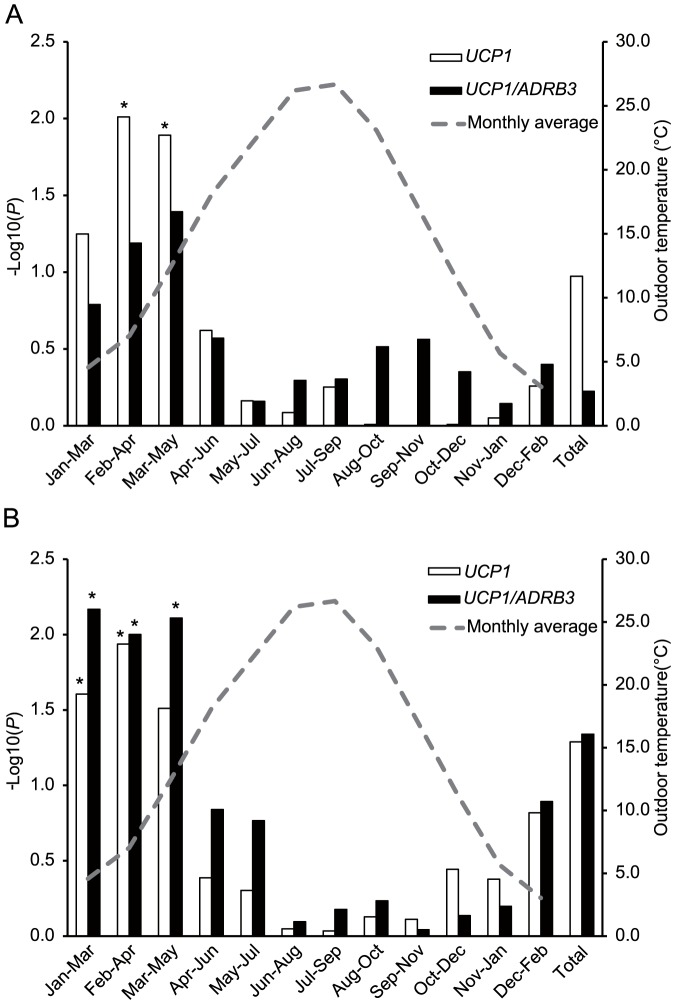
Effects of sampling season on associations with A) VFA and B) VFA adjusted for BMI. The –log of *P*-values for *UCP1* and *UCP1/ADRB3* in individuals sampled in windows of successive 3 months. Average outdoor temperatures in the second month of each 3-month window are shown with a gray broken line. *: *P*<0.05 after applying Bonferroni correction.

**Figure 2:**
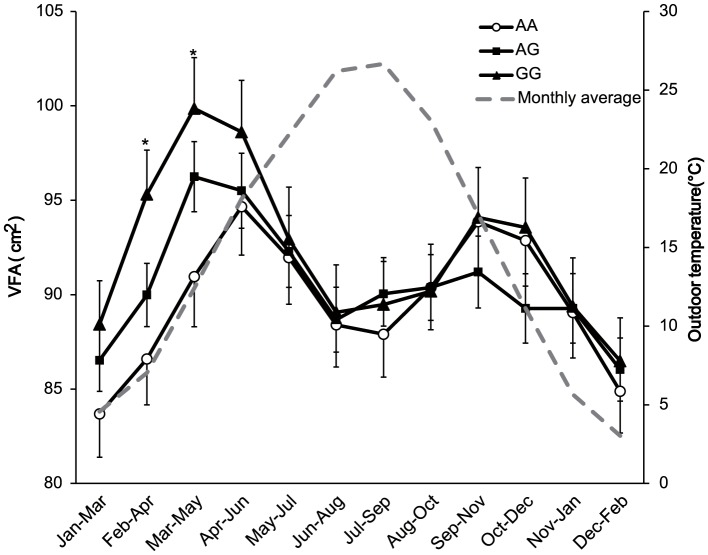
Intergenotype differences in VFA for *UCP1* -3826A/G. For each 3-month window, the means and standard errors of the VFA in the 3 genotype groups are shown. Average outdoor temperatures in the second month of each 3-month window are shown with a gray broken line. Asterisks indicate 3-month windows in which the *UCP1* genotype showed significant associations with VFA.

To more precisely depict the season-dependent effect of *UCP1* on the VFA, associations with VFA were tested in 12 subsets, which each consisted of individuals sampled during overlapping continuous 3-month periods (Figure 1). *UCP1* was significantly associated with VFA in the subsets sampled from February to April and March to May (*P* = 0.0098 and 0.0128, respectively). Association in the subset January to March was marginal (*P* = 0.056). Moreover, significant associations with VFA adjusted for BMI were observed in subsets sampled from January to March and February to April (*P* = 0.0248 and 0.0116, respectively). Subjects sampled from winter to spring generally showed larger VFAs (and VFAs adjusted for BMIs) in comparison with subjects sampled from summer to autumn. Furthermore, the G allele of *UCP1* showed an obvious copy number-dependent increase in VFA among individuals sampled from January to May (Figure 2). For *ADRB3*, the Arg allele tended to associate with a decreased VFA in several subsets, but the association did not survive the multiple testing correction (*P*>0.025). We also tested summed numbers of risk alleles of *UCP1* and *ADRB3* and observed strong associations with VFA adjusted for BMI in subsets sampled from January to May (*P* = 0.0067 ∼ 0.010). *ADRB3* and *UCP1* did not show associations with BMI in all subsets (*P*>0.025).

Next, correlations between the effects of *UCP1* polymorphisms and environmental factors, which possibly account for the seasonality of BAT activity, were tested (Table 3). A set of β coefficients for *UCP1* -3826 G allele, obtained from the linear regression models based on the overlapping 12 subsets (described above), was used as a variable representing temporal difference in the effect of *UCP1*. The average outdoor temperature and average night length of the first month, second (midst) month, third month, and the previous month of each 3-month period (e.g., February, March, April, and January for February–April subset) were used as variables representing environmental factors. β coefficients for G alleles showed negative correlations with average outdoor temperatures of the first month in each subset (Spearman's rank correlation test, *R* = −0.629, *P* = 0.028). Moreover, a stronger correlation was observed for β coefficients and the average outdoor temperature of the previous month (*R* = −0.888, *P* = 0.00011). β coefficients also showed correlation with the average night length of the third month (*R* = −0.643, *P* = 0.024)

**Table 3:** Correlation between the β coefficients for *UCP1* -3826 A/G and the monthly average outdoor temperature/night length.

	*R*	*P*
Average outdoor temperature		
in previous months	−0.888	0.00011
in the first month	−0.629	0.028
in the second month	−0.203	0.527
in the third month	0.224	0.484
Average night length		
in previous months	0.573	0.051
in the first month	0.196	0.542
in the second month	−0.238	0.457
in the third month	−0.643	0.024

Results of Spearman's rank correlation test (*R* and *P* values) are shown. For the definitions of “in previous months”, “in the first month”, “in the second month”, and “in the third month”, see the main text.

## Discussion

Non-shivering thermogenesis by BAT offers resting energy expenditure in adult humans, and therefore, heritable variation of BAT activity is expected to influence the risk for obesity. The *UCP1* -3826 G allele was reported to be associated with reduced BAT activity [8], diminished resting energy expenditure, and reduced thermoregulatory sympathetic nervous system activity [13]. In the present study, we demonstrated the season-dependent effects of *UCP1* -3826A/G on VFA in Japanese adults, in which individuals sampled in cold months but not in hot months showed significant associations. This observation was consistent with the fact that BAT in adult humans required cold exposure for activation and that active BAT was more frequently found in subjects tested in the winter than in the summer [4]. VFA generally tended to be larger in individuals sampled from winter to spring. This could be explained by the reduced physical exercise and/or increased food intake in cold seasons [14]. -3826 A/G is located in a transcriptional enhancer site of *UCP1*, and G allele was shown to reduce the transcriptional activity of UCP1 [15]. G allele was associated with the age-related decrease of BAT activity [8]. During the cold season, G allele carriers would be more vulnerable to visceral fat accumulation due to reduced non-shivering thermogenesis. During the hot season, non-shivering thermogenesis merely contributed to energy expenditure, and *UCP* -3826A/G no longer showed intergenotype differences in VFA. The season-dependent effects of *UCP1*-3826A/G on VFA were supported by the observation that the metabolic response to cold exposure was higher in the winter than in the summer [16]. Our findings also suggested that inconsistency among the previous association studies [6], [7] may be partly explained by the lack of attention to the sampling season.

Although there is no doubt about the crucial role of cold exposure in the activation of BAT, it was still unclear whether ambient temperature was the true factor influencing the seasonality of BAT activity. Au-Yong reported that the seasonality of BAT activity was more closely correlated with night length than with ambient temperature [17]. In the present study, the effects of *UCP1* on VFA were negatively correlated with the average outdoor temperature rather than with night length (Table 3), supporting the predominant role of ambient temperature in activating the BAT. The strongest association was observed in the subsets sampled from February to April. This period did not completely overlap with the coldest months included in the study (Figure 1). Interestingly, the effects of *UCP1* showed a stronger correlation with the average outdoor temperature in the previous month rather than that in sampling months (Table 3). Thus, VFA reduction by cold-induced BAT activation may be manifested after a delay of a few months.

*UCP1* -3826A/G also showed associations with VFA adjusted for BMI. Moreover, *ADRB3* Trp64Arg showed additive effects on the association of *UCP1* and VFA adjusted for BMI. Adjustment for BMI had significant effects on the measurement of visceral fat relative to other organs. We recently identified Tribbles homolog 2 gene (*TRIB2*) as a locus associated with VFA adjusted for BMI in Japanese individuals [11]. Moreover, Fox et al. showed that an intergenic SNP near *TRIB2* was strongly associated with pericardial fat mass [18]. VFA adjusted for BMI may be an estimation of the amount of other ectopic fat depots, which are also metabolically active and can exhibit adverse metabolic abnormalities [19]. The roles of *UCP1* -3826A/G (and possibly *ADRB3* Trp64Arg) on ectopic fat formation should be explored in future studies.

The present findings were obtained from a cross-sectional analysis of single cohort; therefore, a longitudinal analysis and/or a replication study are needed. Interestingly, we found possible sex-specific effects of *UCP1* -3826A/G on VFA (Table S2). Imbalance of sample size does not appear to determine the association since the numbers of males and females were almost even for each analysis (Table 2). Testing the male predominant effect of *UCP1* on VFA reduction would contribute to a debate on the sexual dimorphisms of prevalence and activity of BAT in adult humans [20]. Inter-individual variation in physical activity and BAT activity should be taken into account in future studies. Additionally, it is still uncertain whether the outdoor temperature was the true factor influencing the association of *UCP1* -3826A/G and VFA. Finally, our hypothesis does not consider the role of the *UCP1* in diet-induced thermogenesis [21], which may contribute to resting energy expenditure in a season-independent manner.

In conclusion, *UCP1* -3826A/G was significantly associated with VFA in a season-dependent manner, supporting the importance of cold stress in the activation of BAT and the significance of BAT in the development of obesity in adult humans. Furthermore, association studies of visceral adiposity in adult humans in a geographic region with distinctive seasons might consider employing controls for the sampling season.

## Supporting Information

Table S1 **Effect sizes (β) and *P* values of independent variables in the entire cohort.**
(DOCX)

Table S2 **Sex-specific nature of the effects of *UCP1* -3826 A/G on visceral fat area.**
(DOCX)
