# Supplementary material for: Seasonal Effects of UCP1 Gene Polymorphism on Visceral Fat Accumulation in Japanese Adults
Source: PLoS One. 2013 Sep 25;8(9):e74720. doi: 10.1371/journal.pone.0074720 (PMC3783463; doi:10.1371/journal.pone.0074720)
Supplement: Table S1 — Effect sizes (β) and P values of independent variables in the entire cohort. (DOCX) [file pone.0074720.s001.docx]

Table S1 Effect sizes (β) and *P* values of independent variables in the entire cohort

|  | BMI | | VFA | | a-VFA | |
| --- | --- | --- | --- | --- | --- | --- |
|  | β(S.E.) | *P* | β (S.E.) | *P* | β *(S.E.)* | *P* |
| Sex^1^ | -0.24(0.018) | 3.15E-40 | -0.55(0.014) | 2.2E-273 | -0.40(0.008) | 0 |
| Age | 0.002(0.018) | 0.934 | 0.33(0.014) | 2.3E-111 | 0.32(0.008) | 1.9E-282 |
| Walking speed^2^ | -0.15(0.018) | 1.77E-16 | -0.13(0.014) | 3.2E-19 | -0.03(0.008) | 0.0001 |
| BMI | N.A | N.A | N.A | N.A | 0.63(0.008) | 0 |

BMI: Body mass index, VFA: visceral fat area, a-VFA: VFA adjusted for BMI

^1^Male and female were coded as 0 and 1, respectively.

^2^“Slow” and “Fast” were coded as 0 and 1, respectively.

S.E.: Standard error

N.A.: Not applicable
