# Supplementary material for: Seasonal Effects of UCP1 Gene Polymorphism on Visceral Fat Accumulation in Japanese Adults
Source: PLoS One. 2013 Sep 25;8(9):e74720. doi: 10.1371/journal.pone.0074720 (PMC3783463; doi:10.1371/journal.pone.0074720)
Supplement: Table S2 — Sex-specific nature of the effects of UCP1 -3826 A/G on visceral fat area. (DOCX) [file pone.0074720.s002.docx]

Table S2 Sex-specific nature of the effects of *UCP1* -3826 A/G on visceral fat area

|  |  | VFA | | a-VFA | |
| --- | --- | --- | --- | --- | --- |
|  |  | β(S.E.) | *P* | β(S.E.) | *P* |
| Entire cohort | Females | 0.009 (0.024) | 0.698 | 0.002 (0.011) | 0.851 |
|  | Males | 0.049 (0.023) | 0.037 | 0.027 (0.013) | 0.036 |
| January–April | Females | 0.013 (0.038) | 0.735 | 0.028 (0.018) | 0.192 |
|  | Males | 0.111 (0.040) | 0.005 | 0.015 (0.021) | 0.392 |
| July–October | Females | 0.018 (0.040) | 0.652 | 0.007 (0.040) | 0.685 |
|  | Males | 0.011 (0.017) | 0.784 | 0.010 (0.022) | 0.660 |

β coefficients and *P* values for G allele in the multiple linear regression models (adjusted for age and walking speed) are shown.

S.E.: standard error, VFA: visceral fat area, a-VFA: VFA adjusted for body mass index
